# The mode of action of dimeticone 4% lotion against head lice, *Pediculus capitis*

**DOI:** 10.1186/1471-2210-9-3

**Published:** 2009-02-20

**Authors:** Ian F Burgess

**Affiliations:** 1 Medical Entomology Centre, Insect Research & Development Limited, 6 Quy Court, Colliers Lane, Stow-cum-Quy, Cambridge, CB25 9AU, UK

## Abstract

**Background:**

Treatment of head lice using physically acting preparations based on silicones is currently replacing insecticide use due to widespread resistance to neurotoxic agents. It has been postulated that some products act by asphyxiation, although the limited experimental evidence and the anatomy of the louse respiratory system suggest this is unlikely.

**Results:**

Observation over several hours of lice treated using 4% high molecular weight dimeticone in a volatile silicone base showed that, although rapidly immobilised initially, the insects still exhibited small movements of extremities and death was delayed. One common effect of treatment is inhibition of the louse's ability to excrete water by transpiration through the spiracles. Inability to excrete water that is ingested as part of the louse blood meal appears to subject the louse gut to osmotic stress resulting in rupture. Scanning electron microscopy coupled with X-ray microanalysis to detect silicon showed dimeticone lotion is deposited in the spiracles and distal region of the tracheae of lice and in some cases blocks the lumen or opening entirely.

**Conclusion:**

This work raises doubts that lice treated using dimeticone preparations die from anoxia despite blockage of the outer respiratory tract because movements can be observed for hours after exposure. However, the blockage inhibits water excretion, which causes physiological stress that leads to death either through prolonged immobilisation or, in some cases, disruption of internal organs such as the gut.

## Background

Resistance of head lice, *Pediculus capitis*, to neurotoxic insecticides such as the pyrethroids and malathion has become widespread in developed countries since the first reports in the early 1990s. [1-3] The impact of resistance on therapy has had several effects including: repeated exposure of children to insecticides with an increasing reluctance on the part of parents to use pesticide based products [4]; increased prevalence of lice in most communities [5]; and a plethora of products available through online purchase that claim efficacy but without clinical evidence.

Dimeticone 4% lotion is a recent new product development in treatment of head louse infestation. [6] From the product conception it was recognised that its mode of action was to kill the insects by physical mechanisms rather than by neurotoxicity. However, the mode of action of the product has been unclear and other investigators working with dimeticone preparations of different molecular weight, as well as those investigating more straightforward occlusive creams, have loosely referred to the mode of action of those products as "asphyxiation" or "suffocation" but without clear biological evidence in support of their claims. [7,8]

The structure of the louse respiratory system minimises the risk of penetration by fluids so demonstration that a product is capable of entering the respiratory tract of a louse is not adequate evidence for anoxic asphyxiation. [9] Additionally it has long been recorded that lice are able to survive immersion in water for several hours, presumably tolerating long periods without oxygen, and specific experimental investigation of the potential for suffocation by various preparations has not been shown to be currently feasible. [10]

Dimeticone 4% lotion is a licensed medicinal product in the UK and a class I medical device in most other European countries. It contains 4% w/w 100000 centistokes (CSt) dimeticone and 96% w/w cyclomethicone D5. Dimeticone is a polydimethylsiloxane with the chemical formula  (CH_3_)_3_SiO [SiO(CH_3_)_2_]_n_Si(CH_3_)_3_, where *n *is the number of repeating monomer  units [SiO(CH_3_)_2_], which is directly proportional to the molecular weight and viscosity. In the case of 100000 CSt dimeticone the average value of *n *is 1875, giving an average molecular weight of 139050. [11] Cyclomethicone D5 is the volatile cyclic silicone, decamethylcyclopentasiloxane.

Head lice feed exclusively on blood and take several feeds each day. However, they do not excrete liquid urine or faeces like other blood feeding insects. Evidence from our laboratory (unpublished observations) shows that most water ingested as blood is excreted via the spiracles and starved lice die from dehydration within a relatively short time. This appears to occur in two stages. During the first, the lice show little physical change, are able to feed if given the opportunity, but the abdomen may be seen to decrease in volume. At the second stage, which is reached at any time between 3 and 24 hours following their previous meal, the lice remain capable of walking, will fasten to the skin and attempt to suck blood, but are unable to feed, presumably because they are too dehydrated to produce the saliva needed to prevent haemostasis. Complete immobility and death may occur at any point after this. Other investigators have found a mean time to immobility of 21.3 ± 12.1 hours. [12,13]

Early laboratory investigation of the effect of dimeticone 4% lotion on lice suggested it was capable of entering the spiracles and tracheae with resultant disruption of physiological mechanisms such as water management, resulting in gut rupture of recently fed lice. [6] However, other investigators, using a different commercially available mixture of low molecular weight dimeticone compounds, have questioned this interpretation, preferring to attribute the activity of silicone based lotions to suffocation, despite the high tolerance of lice for anoxic effects and the high oxygen permeability of silicones. [8]. I have conducted an investigation of the physical distribution of high molecular weight dimeticone 4% lotion when applied to head lice, and of the physiological outcome of treatment, as a contribution towards understanding the activity of this type of product.

## Methods

### Insects and treatments

Head lice, *Pediculus capitis*, were obtained by combing infested children from whom assent and parental consent had been obtained under ethical approval from Cambridgeshire 3 (formerly Peterborough & Fenland) Research Ethics Committee.

Lice for observation of physiological effects were treated using silicone lotions, either by immersion or else by application of droplets to the cuticle surface using a micro-syringe and needle. Silicone lotions used in these experiments were Hedrin^® ^4% dimeticone lotion (Thornton & Ross Ltd, Huddersfield, UK), which contains 4% high molecular weight dimeticone, and Nyda^® ^L (G. Pohl-Boskamp GmbH & Co. KG, Hohenlockstedt, Germany), an approximately 92% silicone content mixture of two low molecular weight dimeticones in equal proportions.

Lice for weight loss experiments were weighed individually using a *Sartorius 4503 *micro-balance (maximum tare 4.1 grams, d = 0.001 milligrams). Each louse was weighed, fed on the back of a volunteer hand, weighed again, and then at 15 minute intervals. Some fed lice were immersed in dimeticone 4% lotion for 10 seconds immediately after feeding then blotted on absorbent tissue to remove fluid before subsequent weighing.

### Electron microscopy

Lice for electron microscopy were treated using dimeticone 4% lotion applied by immersion of the insects for 10 seconds followed by draining of excess fluid on Whatman No 1 filter paper.

Surface micrographs of the lice were obtained using a FEI/Philips XL30 ESEM environmental scanning electron microscope operating in wet mode i.e. using water vapour as an imaging gas.

The analysis of the chemical elements found in louse spiracles was achieved by using a Quanta 200 3D dual beam scanning electron microscope equipped with a Gatan Cryotransfer System and an Oxford Instruments INCA X-ray microanalysis package.

The specimens were attached to a sample stub using Tissue-Tek^® ^embedding fluid, quickly frozen to preserve their integrity by plunging into liquid nitrogen slush, and then platinum coated and viewed at -180°Celsius.

Internal structures of spiracles were investigated by milling away tissue at the site of interest using a gallium, focussed ion beam (FIB). This allowed the chemical elements found within the structure to be qualitatively identified using the X-ray analysis system. The key marker element used for dimeticone 4% lotion was silicon as it is not found at any significant background levels in lice.

## Results

### Physical and physiological effects of silicone treatment

Lice immersed in silicone lotions were observed to cease overt mobility within 60 seconds, as reported previously by different observers [6,8,14,15]. Lice removed from the either of the silicone fluids exhibited no signs of recovery after blotting or if washed with shampoo and water to remove surface silicone. Lice left in the fluids for longer periods also remained motionless, with the exception of occasional peristaltic movements of the gut. However, after about 2 hours some lice immersed in low molecular weight dimeticone began to exhibit small movements of limbs, such as flexing of the tibiae or tarsae, as well as small movements of the antennae, indicating that lice immersed in the fluid had not suffered anoxic asphyxiation within that period of time. Movements of this type were seen to continue for more than 1 hour after first being noted.

Richling and Bökeler [8] reported rapid flow of the low molecular weight silicone mixture into the tracheae of immersed lice and entry of silicone fluid into tracheae when applied to the surface of the louse abdomen using a micro-syringe. [14] I attempted to repeat these experiments on several occasions, using more than 50 lice of different ages and development stages. Neither of the silicone lotions, when applied in this way, could be induced to spread through the tracheal system of a louse beyond the junction of the trachea and the spiracle.

**Figure 1:**
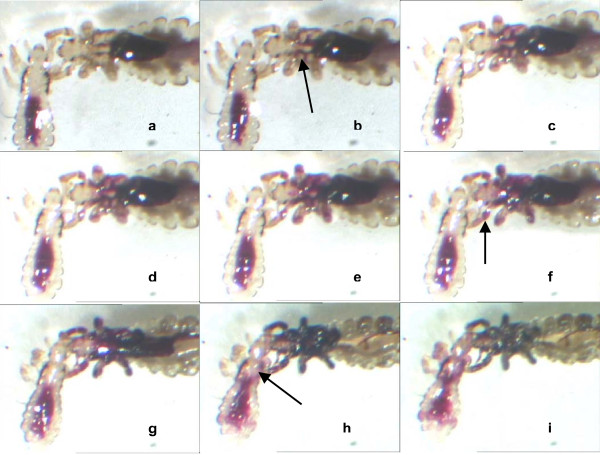
**Effect of dimeticone 4% lotion on the integrity of the gut in head lice**. This sequence of images was taken from a continuous video observation of head lice treated with dimeticone 4% lotion over a period of 17 hours. The gut of the larger louse first showed signs of rupture approximately 5 hours after treatment (arrowed, image b), with blood release over 2–3 hours spreading throughout the thorax and into the limbs (arrowed, image f). The small louse's gut ruptured approximately 14.5 hours after treatment with seepage into the thorax (arrowed, image h). After 17 hours the larger louse appeared dehydrated (image i).

However, when recently fed lice were treated and left in contact with dimeticone 4% lotion it was found that some experienced gut rupture after several hours of contact with the fluid. These insects were mostly found to show no obvious physical changes for between 12 and 17 hours, but continuous video monitoring during this period detected the onset and spread of blood from the midgut into and throughout the haemocoel of the thorax and head following rupture of the gut wall (Figure 1). In this series of images, the smaller louse, which had ingested less blood, suffered gut rupture rather later than the more replete larger louse, presumably because with a lower fluid volume it was able to tolerate any stresses for a longer period. The gut in some fed lice and most unfed lice was found to retain its integrity. Death of these insects was assumed to be entirely related to prolonged immobilisation preventing access to food.

**Figure 2:**
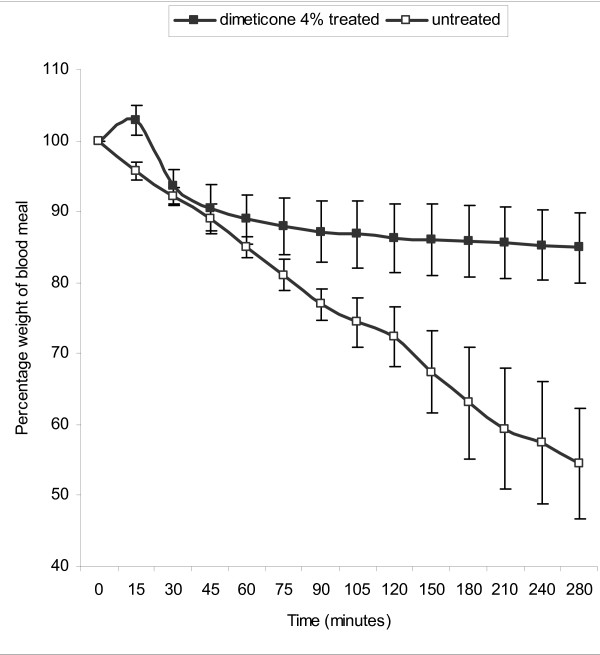
**Effect of dimeticone 4% lotion on water loss from fed lice**. Head lice were weighed prior to feeding, immediately after feeding to determine the blood meal weight, and then at regular intervals. This graph compares weight loss in lice treated by immersion in dimeticone 4% lotion immediately after feeding with weight loss in untreated lice, to show the inhibition of water loss by transpiration following treatment. The initial rise in weight of treated lice was due to silicone fluid residues on the cuticle that evaporated over the next 30 minutes.

Evidence that dimeticone 4% lotion treatment inhibits water loss by lice was obtained by weighing. Untreated fed lice showed a steady loss of weight during the period after feeding (Figure 2). Fed lice treated with dimeticone 4% lotion initially showed an increase in weight due to the surface coating of silicone fluid. However, a rapid reduction in weight then occurred during the first 30 minutes, as the volatile cyclomethicone evaporated, followed by a sustained reduction in weight loss thereafter. Between 45 minutes and 280 minutes after treatment the dimeticone 4% treated group had lost a mean of 5.6% of the weight of the ingested blood meal (15.1% overall) compared with a mean loss of 34.6% over the same period (45.6% of the blood meal overall) in the untreated group (Figure 2).

### Scanning electron microscopy

The external physical structures of both thoracic and abdominal spiracles, shown by SEM, and the internal structure of the abdominal spiracle, revealed by milling, were found to be essentially the same as described from light microscope histology by Webb [9]. Thoracic and abdominal spiracles differ in external appearance and in details of internal structure but are essentially similar. We concentrated our investigation mainly on abdominal spiracles.

**Figure 3:**
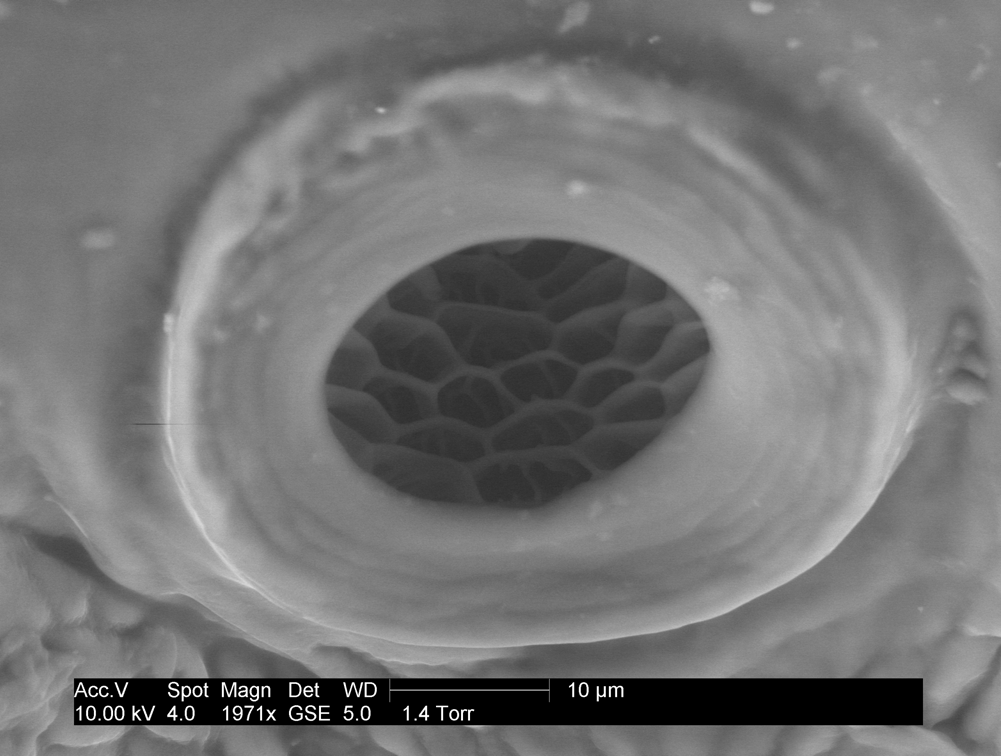
**Untreated thoracic spiracle**. SEM of an untreated thoracic spiracle to show the complex pattern of plates and chambers within the atrium.

Each abdominal spiracle is surrounded by a sclerotised area of cuticle known as a paratergal plate. The thoracic cuticle is highly sclerotised throughout. Each spiracle has a fixed opening surrounded by a discoidal collar, the peritreme, which is fixed on the thorax but partially free at the margins on the abdomen spiracles. The thoracic spiracle is simpler in construction with an atrial chamber lined by a series of cuticle plates that form ledges and chambers in a "honeycomb" like arrangement (Figure 3). On the abdomen the atrium is more complex in that the distal section is expanded laterally to form a mushroom shaped chamber. Both types of spiracle constrict in the proximal portion to form a narrow duct, which is described by Webb [9] as passing through a mass of tissue, the spiracular gland, before entering the trachea.

**Figure 4:**
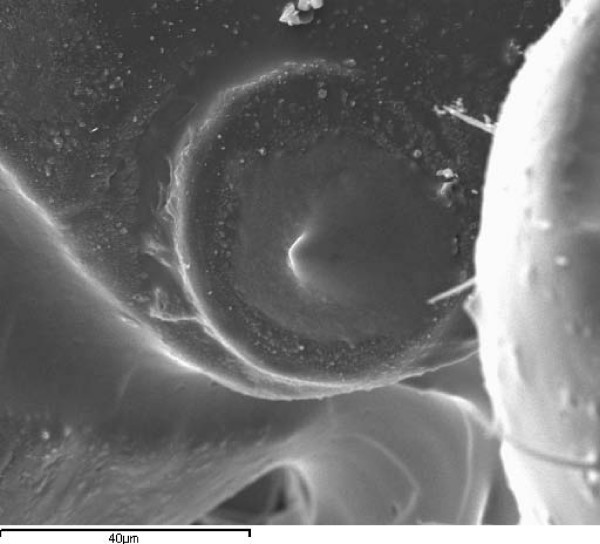
**Treated abdominal spiracle occluded by a silicone plug**. SEM of a treated spiracle in which the whole of the aperture is blocked by a silicone deposit. X-ray analysis showed that elemental silicon was present on the surrounding cuticle of the body, the peritreme of the spiracle, and the occluding plug across the spiracle opening.

**Figure 5:**
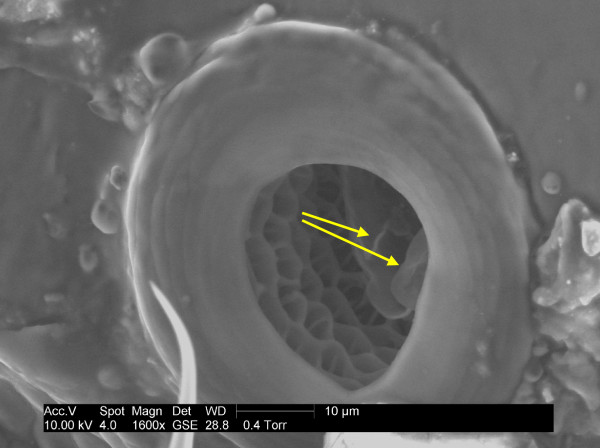
**Untreated thoracic spiracle showing secreted material**. SEM of an untreated spiracle to show a deposit within the atrium of secreted material of louse origin (arrowed). X-ray analysis showed this deposit was free from silicon.

Using high molecular weight dimeticone lotion we were able to detect that deposits of silicone material formed a coating inside the spiracle atrium, or even in some cases blocking the spiracle opening entirely (Figure 4). These deposits were superficially similar in appearance to louse derived secretion that was seen in some spiracles of untreated lice (Figure 5) but it was possible to distinguish them from material of louse origin by detection of high levels of elemental silicon in the deposit, whereas louse secretions and cuticle were devoid of silicon.

**Figure 6:**
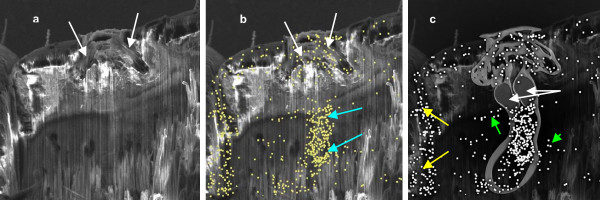
**Demonstration of deposited silicon in the trachea after treatment with dimeticone 4% lotion**. SEM sequence of a treated abdominal spiracle after gallium ion beam milling. (a) Vertical section through the complex, mushroom shaped atrium with its "honeycomb-like" pattern of plates and chambers (arrowed). (b) The superimposed X-ray spectrograph selecting for presence of silicon. Each dot represents a single reading from the sequential scan. A high concentration of silicon responses can be seen in the atrium of the spiracle (white arrows) and in the distal region of the trachea (blue arrows). (c) Diagram to show the structure of the spiracle, spiracular glands (white arrows), and distal trachea, based on diagrams produced by Webb [9] superimposed on the silicon scan. The high intensity of silicon response on the left of the image (yellow arrows) is due to silicone deposited on the surface of the cuticle, which was cut through at that point. Some silicon can be seen in non-respiratory tract tissues (green arrows) due to scattering of silicone fragments, from deposits on the cuticle surface, by the beam of gallium ions during the milling process.

X-ray analysis of the interior of the spiracle atrium, after milling away the surrounding tissues, revealed deposits of silicon throughout the atrium area and the distal (outermost) section of the trachea (Figure 6). This shows that the lotion containing 4% high molecular weight dimeticone had penetrated throughout the spiracle structure, with a greater concentration in the region close to the spiracular gland and the outermost section of the trachea.

## Discussion

X-ray spectroscopy linked to scanning electron microscopy shows that a head louse treatment lotion product containing 4% high molecular weight dimeticone is able to enter and form a coating to the internal surfaces of the spiracles of lice. This coating occludes the internal structures of the spiracle and blocks the opening of the trachea. Lice soaked with dimeticone 4% lotion experience physical disruption of the gut several hours after treatment.

In commenting on the unusual structure of the spiracles of lice of the genus *Pediculus*, Webb [9] stated, "The passage of air from the atrium through the narrow duct of the spiracular gland in itself seems so difficult that any muscles designed to close this aperture further, even if the duct is not filled with secretion, would appear to be unnecessary. On the other hand it is possible that contraction of the muscle forces the trachea against the spiracular gland and causes the duct to gape thus enlarging the aperture between the atrium and the trachea. If this is so then contraction of the muscle opens the spiracle, a condition which appears to be in contrast to that obtaining in other Anoplura."

Webb's description and conclusions contradict the description given by Buxton in his monograph on human lice, "There are six pairs of abdominal spiracles, carried on segments 3 to 8. All spiracles (including that on the thorax) possess a closing apparatus, which appears to work by compressing or buckling the trachea a short distance below the surface of the body. It seems highly probable that the louse uses this in order to reduce the loss of water from within its respiratory system." [16] This statement, based on drawings by Hase, [17] has become the "received wisdom" about the mechanism of action of the human louse respiratory apparatus, in that it is often stated that when immersed in water they "close their spiracles" and enter a state of immobility. [18,19] The actual mechanism operating is important because blockage of spiracles, or relaxation of muscles that close the tracheae, forms the basis of the proposed "asphyxiation" mode of action for several newly developed products that claim physical activity against lice. [6,7,20,21]

If lice immersed in water "close" their respiratory tracts in order to exclude fluid it presumably requires considerable anaerobic metabolic energy expenditure to continuously contract the muscles closing the tracheae, which may not be sustainable for the length of time lice can remain viable while immersed in water. However, if the tracheae are normally "closed" and only "open" to allow occasional air intake, like many terrestrial insects, [22,23] it would certainly explain the ability of lice to readily exclude water when immersed. However, as cyclic and discontinuous gas exchange patterns are also considered methods whereby insects limit water loss, the evidence we have from weighing experiments appears to suggest that, after feeding at least, lice expel water freely rather than conserve it, and the stress induced when water loss is prevented indicates that louse physiology is not like that of most other insects. In some ways louse respiratory behaviour is more consistent with the proposal that oxygen is relatively toxic for insects so opening of the spiracles is limited and mainly used for excretion of carbon dioxide. [24,25]

If the normal anatomical and physiological operation of the louse respiratory structures is to have a closed trachea it is difficult to see how even a low surface tension fluid such as low viscosity silicone could penetrate deeply into the trachea. Richling and Böckler [8] applied small drops of fluid to the tip of the abdomen, which allowed silicone to systematically flow through the tracheal system and "vent" through untreated spiracles. When an insect is immersed in or coated with dimeticone lotion, the fluid would enter all spiracles equally and trap most of the air inside, in a similar manner to the way water blocks the tracheoles but does not enter the alveoli of the lungs in drowned mammals. However, it is not difficult to conceive how in lice the entrance to this set of structures can be physically blocked by high molecular weight dimeticone deposited from a low molecular weight volatile solvent, as demonstrated in this study. Furthermore, if lice live continuously with a low oxygen requirement it is unlikely that even if deep penetration of the tracheae by silicone fluids did occur it would result in anoxic asphyxiation as the primary cause of death, because silicone fluids have a high permeability to oxygen. Therefore, the most likely mode of action of dimeticone 4% lotion (Hedrin^® ^4% lotion) against head lice is to permanently physically block the outermost sections of the louse respiratory tract, preventing water excretion, which may lead to gut rupture in recently fed insects and irreversible immobilisation, leading ultimately to death, in others.

## Conclusion

Silicone based products for treatment of head louse infestation show an immediate immobilisation effect on the insects that mimics immersion in water. However, because silicones are difficult to remove or wash off, lice do not recover as they would if removed from water but remain immobile until death.Contrary to the widespread opinion that physically acting pediculicides work by suffocation the evidence is that occlusion of spiracles and tracheal trunks by silicone deposits produces a barrier that inhibits excretion of water, which results in osmotic stress leading ultimately to gut rupture in fed insects.
